# TO WHAT DEGREE IS AGEISM ACTUALLY DISABLEISM? A QUALITATIVE STUDY

**DOI:** 10.1093/geroni/igz038.2111

**Published:** 2019-11-08

**Authors:** Mariska van der Horst, Sarah Vickerstaff

**Affiliations:** 1 VU Amsterdam, Amsterdam, Netherlands; 2 University of Kent, Canterbury, England, United Kingdom

## Abstract

Ageism has been identified as a possible threat to the extending working lives agenda that is prevalent in many Western countries. It recently became a popular topic of research, but is not yet well understood. In this article we explore to what degree ageism is actually hidden disableism. We suggest that not all ageism is likely to be disableism, but a large part is, with the difference that it is not (necessarily) about actual impairments, but expected impairments. Using data from a large case study based project in the UK (containing 185 participants), we further assess to what degree older workers link ageism to disableism in their own accounts of future work plans. We conclude that even though we would still expect ageism to affect employment of older workers, without disableism it is unlikely that ageism would be as detrimental to the employment of older workers as it is now.

